# Soybean Resistance to White Mold: Evaluation of Soybean Germplasm Under Different Conditions and Validation of QTL

**DOI:** 10.3389/fpls.2018.00505

**Published:** 2018-04-20

**Authors:** Ramkrishna Kandel, Charles Y. Chen, Craig R. Grau, Ann E. Dorrance, Jean Q. Liu, Yang Wang, Dechun Wang

**Affiliations:** ^1^Horticultural Sciences Department, University of Florida, Gainesville, FL, United States; ^2^Department of Crop, Soil, and Environmental Sciences, Auburn University, Auburn, AL, United States; ^3^Department of Plant Pathology, University of Wisconsin, Madison, WI, United States; ^4^Department of Plant Pathology, Ohio Agricultural Research and Development Center (OARDC), The Ohio State University, Wooster, OH, United States; ^5^Pioneer Hi-Bred International, Inc., Johnston, IA, United States; ^6^Soybean Research Center, Jilin Academy of Agricultural Sciences, Changchun, China; ^7^Department of Plant, Soil and Microbial Sciences, Michigan State University, East Lansing, MI, United States

**Keywords:** Sclerotinia stem rot, soybean white mold, greenhouse inoculation, prediction of field resistance, validation of QTL for soybean white mold resistance, drop-mycelium, spray-mycelium, *Sclerotinia sclerotiorum*

## Abstract

Soybean (*Glycine max* L. Merr.) white mold (SWM), caused by *Sclerotinia sclerotiorum* (Lib) de Barry), is a devastating fungal disease in the Upper Midwest of the United States and southern Canada. Various methods exist to evaluate for SWM resistance and many quantitative trait loci (QTL) with minor effect governing SWM resistance have been identified in prior studies. This study aimed to predict field resistance to SWM using low-cost and efficient greenhouse inoculation methods and to confirm the QTL reported in previous studies. Three related but independent studies were conducted in the field, greenhouse, and laboratory to evaluate for SWM resistance. The first study evaluated 66 soybean plant introductions (PIs) with known field resistance to SWM using the greenhouse drop-mycelium inoculation method. These 66 PIs were significantly (*P* < 0.043) different for resistance to SWM. However, year was highly significant (*P* < 0.00001), while PI x year interaction was not significant (*P* < 0.623). The second study compared plant mortality (PM) of 35 soybean breeding lines or varieties in greenhouse inoculation methods with disease severity index (DSI) in field evaluations. Moderate correlation (r) between PM under drop-mycelium method and DSI in field trials (*r* = 0.65, *p* < 0.0001*)* was obtained. The PM under spray-mycelium was also correlated significantly with DSI from field trials (*r* = 0.51, *p* < 0.0018*)*. Likewise, significant correlation (*r* = 0.62, *p* < 0.0001) was obtained between PM across greenhouse inoculation methods and DSI across field trials. These findings suggest that greenhouse inoculation methods could predict the field resistance to SWM. The third study attempted to validate 33 QTL reported in prior studies using seven populations that comprised a total of 392 F_4 : 6_ lines derived from crosses involving a partially resistant cultivar “Skylla,” five partially resistant PIs, and a known susceptible cultivar “E00290.” The estimates of broad-sense heritability (*h*^2^) ranged from 0.39 to 0.66 in the populations. Of the seven populations, four had *h*^2^ estimates that were significantly different from zero (*p* < 0.05). Single marker analysis across populations and inoculation methods identified 11 significant SSRs (*p* < 0.05) corresponding to 10 QTL identified by prior studies. Thus, these five new PIs could be used as new sources of resistant alleles to develop SWM resistant commercial cultivars.

## Introduction

Soybean white mold (SWM), caused by *Sclerotinia sclerotiorum* (Lib) de Bary, is a major soybean (*Glycine max* L. Merr.) disease in the Upper Midwest region of the United States and southern Canada. The SWM is the fourth most important cause of yield loss based on the estimated yield loss from the top 28 US soybean-producing states (Koenning and Wrather, 2010). However, the progress in the development of resistant cultivars is very slow due to the quantitative nature of the disease resistance (Kim and Diers, 2000; Arahana et al., 2001; Peltier et al., 2012) and lack of certainty to achieve desired SWM pressure during field evaluations of the breeding materials.

*Sclerotinia sclerotiorum* overwinters in the soil and debris as resting structures called sclerotia (Yang et al., 1998). However, infections of soybean in the field environments are caused by ascospores that first land on the delicate plant parts such as flower petal. Ascospores colonize them, and then progress downward by infecting and girdling the main stem leading to eventual plant death. The typical symptoms of diseased plants include necrotic leaves, bleached lesions on stems and pods, white fluffy mycelial growth, and presence of black sclerotia on the leaves, stems, and pods (Chen and Wang, 2005). Wet soil and canopy conditions at flowering favor the development of the disease (Grau, 1988). Irrigation, narrow plant spacing, early flowering, and thick vegetation contribute to the development and spread of SWM (Boland and Hall, 1987; Kim et al., 2000). Control of the disease through chemical means has been proven difficult because it demands several preventive and systemic treatments (Mueller et al., 2004). Indeed, control through chemical approaches has the added risk of increased production costs.

Host plant resistance (HPR) plays a key role in effective management of the disease (Kim and Diers, 2000; Kurle et al., 2001). However, determination of true physiological resistance to SWM in field conditions is confounded by many factors, namely, plant density, canopy architecture, flowering date, and maturity (Nelson et al., 1991; Kim et al., 1999). In fact, Kim and Diers (2000) identified two QTL for resistance to SWM but they could be rather involved in disease avoidance than in real resistance because of their significant association with disease avoidance phenotypes (disease klendusity), viz., flowering time, plant height and lodging. These structural disease avoidance phenotypes (Boland and Hall, 1987; Kim and Diers, 2000; Rousseau et al., 2004) coupled with complex genetic and environmental interactions complicate the determination of true physiological resistance in field trials (McCaghey et al., 2017). Moreover, reactions of soybean genotypes varied when isolates of *S. sclerotiorum* with varying levels of aggressiveness were used for disease screening (McCaghey et al., 2017; Willbur et al., 2017).

Various studies have attempted to unravel the underlying genetics of SWM resistance using field, lab, and greenhouse inoculation methods. Three, 28, seven, four, and three SWM resistance QTL were identified respectively by Kim and Diers (2000), Arahana et al. (2001), Guo et al. (2008), Vuong et al. (2008), and Huynh et al. (2010). Lately, genome-wide association (GWAS) and epistatic (GWES) studies have revealed single-nucleotide polymorphisms (SNPs) associated with SWM resistance (Bastien et al., 2014; Iquira et al., 2015; Zhao et al., 2015; McCaghey et al., 2017; Moellers et al., 2017; Wei et al., 2017). Moellers et al. (2017) identified 58 main effect loci and 24 epistatic interactions associated with SWM resistance.

Although HPR is the most cost-effective method of SWM control, the progress in development of soybean cultivars has been slow, due, in part, to the unavailability of reliable methods to evaluate disease responses. There exist numerous greenhouse and laboratory inoculation methods to evaluate SWM resistance, namely, colonized oat kernels inserted into cotyledons (Grau and Bissonette, 1974), excised stem or detached leaf assay (Chun et al., 1987; Leone and Tonneijck, 1990; Nelson et al., 1991; Miklas et al., 1992; Steadman et al., 2001; Kull et al., 2003; Vuong et al., 2004), cut stem inoculation (Kull et al., 2003; Vuong et al., 2004), oxalic acid assay (Noyes and Hancock, 1981; Tu, 1989; Kolkman and Kelly, 2000), and cut-petiole inoculation (Del Rio et al., 2001). Most of these methods had low to moderate correlation values between greenhouse and field data and in fact, are also very tedious to apply in large scale (Boland and Hall, 1987; Chun et al., 1987; Nelson et al., 1991; Kim et al., 2000). No correlation was found between data from field trials and a limited-term-inoculation method in greenhouse evaluations (Boland and Hall, 1987). Of the eight correlations between excised stem method in the laboratory and field evaluations, only one showed statistically significant relationship (Chun et al., 1987). Similarly, no significant correlations were observed between excised stem method in the laboratory and field evaluations (Nelson et al., 1991). However, field artificial inoculation methods currently available do not guarantee adequate and homogeneous spread of the inoculum because of unpredictable environmental conditions and overlap of unequal natural infection across genotypes. Lack of certainty in achieving desired disease pressure in field trials also contribute to observed field resistance. It thus calls for controlled greenhouse evaluations to determine the true physiological resistance.

Further, results from greenhouse evaluations are difficult to apply in field trials because most of the greenhouse evaluation techniques are time-consuming and suffer from low efficiency, low reproducibility, and high cost. In light of these concerns, Chen and Wang (2005) developed two convenient methods to screen for SWM resistance using whole-plant inoculation. Botha et al. (2009) compared six greenhouse inoculation techniques (spray-mycelium, drop-mycelium, cut stem, cotyledon, straw, and petiole) to screen for SWM resistance. Spray-mycelium method proved to be most effective and reliable technique among them to consistently induce the highest disease incidence and severities. However, the suitability of greenhouse evaluations based on spray- and drop-mycelium techniques for predicting field resistance to SWM has not been proved yet.

At the beginning of this study, a total of 33 QTL associated with SWM resistance were reported (Kim and Diers, 2000; Arahana et al., 2001; Guo et al., 2008). The objectives of this study were to: (a) evaluate the reactions of 66 partially resistant PIs from Hoffman et al. (2002) using drop-mycelium inoculation method, (b) predict field resistance to SWM using greenhouse inoculation methods, and (c) validate the 33 QTL reported in three prior studies using seven populations comprising 392 F_4:6_ lines derived from crosses involving a well-known resistant cultivar, a susceptible cultivar, and five partially resistant PIs.

## Materials and methods

### Plant materials

A total of 66 PIs, 35 breeding lines or varieties, and 392 F_4 : 6_ lines were used in three independent studies. The first study investigated 66 PIs that were selected from over 6,000 PIs based on their partial resistance to SWM in the fields and greenhouse evaluations (Hoffman et al., 2002). These PIs belong to maturity groups from 0 to IV. The second study consisted of 35 soybean breeding lines or varieties that were chosen from different north-central soybean breeding programs based on the availability of their phenotypic data for reaction to SWM. The third study used 392 F_4 : 6_ lines (constituting seven populations) that were derived from seven crosses involving a partially resistant variety “Skylla,” five partially resistant PIs reported by Hoffman et al. (2002), and a known susceptible line “E00290” (Table 1). Skylla carries resistance to SWM from a cultivar “NKS 19-90” (Wang et al., 2006). NKS 19–90 has partial resistance to SWM (Kim et al., 1999) and is reported to harbor SWM resistance QTL (Kim and Diers, 2000; Arahana et al., 2001). The five PIs: PI 89001, PI 153259, PI 437764, PI 548404, and PI 548312 were reported to have resistance level comparable to that of NKS 19–90 (Hoffman et al., 2002).

**Table 1:** Seven populations (392 F_4 : 6_ lines) derived from new PI resistance sources and their reactions to soybean white mold (SWM) based on mean plant mortality from two greenhouse evaluation methods (spray-mycelium and drop-mycelium).

**Population**	**Female parent**	**Male parent**	**Number of lines**	**Plant mortality**	**σ^2^ G ± SE^d^**	***h*^2^ ± SE^e^**
1	E00290^a^ (73.0)	PI 89001	59	45.7	137.2 ± 41.5	0.64 ± 0.21
2	E00290	PI 437764 (57.5)	50	45.4	176.5 ± 61.8	0.66 ± 0.22
3	E00290	PI 548312^c^ (24.6)	63	63.5	59.5 ± 29.4	0.39 ± 0.19
4	Skylla^b^	PI 89001^*c*^	62	48.8	137.2 ± 50.8	0.52 ± 0.19
5	Skylla (30.0)	PI 153259^c^ (62.0)	51	40.7	62.3 ± 37.2^*^	
6	Skylla	PI 437764^c^	38	48.6	72.2 ± 47.0^*^	
7	Skylla	PI 548404^c^ (61.0)	69	29.0	45.8 ± 33.3^*^	

*In parentheses are mean plant mortality for parents*.

a *Susceptible to soybean white mold (SWM)*.

b *Carries resistance to soybean white mold (SWM) from NKS19-90*.

c *Partially resistant to soybean white mold (SWM) (Hoffman et al., 2002)*.

d *Estimates of genotypic variance ±standard errors*.

e *Estimates of broad-sense heritability ±standard errors*.

* *Non-significant estimates of genotypic variance*.

### Greenhouse experimental design

Six seeds per genotype and replication were planted in a 10 × 10 × 15 cm plastic pot. The pots were arranged in a randomized complete block design (RCBD). NKS 19–90 and Olympus were used as resistant and susceptible checks, respectively for the 66 PIs and the 392 F_4 : 6_ lines, whereas NKS 19–90 and BSR101 served as resistant and susceptible checks, respectively for the 35 breeding lines or varieties. Plants were allowed to germinate and reach a V3 growth stage (Fehr and Caviness, 1977) before inoculation was performed. Plants were watered adequately during the entire experiment. A 32-ounce clear plastic PET cups with bottoms removed were placed upside down over all pots to ensure upright architecture of plants, which in turn facilitated an infection point on the top of the plant and then downward progression of fungal growth.

### Greenhouse inoculations

The 66 PIs were evaluated by the drop-mycelium method only (Chen and Wang, 2005) using three replications each in the winter of 2009 and 2010, whereas the 35 breeding lines or varieties (Table 2) were evaluated by both the drop-mycelium and the spray-mycelium methods (Chen and Wang, 2005) using three replications in May, October, and December of 2005 and winter of 2010, respectively. Three-hundred and ninety-two F_4 : 6_ lines were also evaluated by both the spray-mycelium and the drop-mycelium methods using three replications. The experiments were conducted in the greenhouse at Michigan State University. The *S. sclerotiorum* isolate 105HT provided by Dr. Glen Hartman at USDA-ARS was used in the greenhouse evaluations. The procedures for the greenhouse evaluations were as described by Chen and Wang (2005). Briefly, fungal inoculums were prepared from the sclerotia obtained from the previous year. The sterilized sclerotia were grown in potato dextrose agar (PDA) medium for 3–4 days and then re-cultured into a new PDA to keep the stock fresh. The mycelia were cut into small pieces and transferred into liquid potato dextrose broth (PDB). The PDB was homogenized by constantly shaking at a speed of 200 rpm on a G10 GYROTORY shaker (Edison, NJ) for four nights. The mycelium suspension was homogenized in a household blender just before inoculation to ensure uniformity in mycelium. The blended mycelium suspension was evenly sprayed on the leaves at approximately 4.6 ml/per plant at the V3 growth stage by a battery-operated hand sprayer. Similarly, the homogenized mycelium suspension was applied onto the apical meristem of plants at approximately 1 ml/plant with a washer bottle at V3 growth stage in drop-mycelium method. The inoculated plants were placed in plastic chambers, which had two humidifiers at opposite ends of each chamber to maintain a near 100% humidity inside the chambers. The plastic chambers consisted of two benches canopied by a semi-opaque plastic. The humidifiers were set to a 2 min on and 3 min off regime 24 h a day. Approximately 7 to 10 days after inoculation when the susceptible checks had reached approximately over 70% mortality, the total number of dead plants were counted, and the plant mortality was calculated as follows; plant mortality (PM) = number of dead plants/total number of plants in a pot.

**Table 2:** Reaction of the 35 soybean breeding lines or varieties to soybean white mold (SWM) as measured by plant mortality (PM) in the greenhouse inoculations (drop- and spray-mycelium methods) and disease severity index (DSI) in Iowa and Wisconsin field trials.

**PM^a^**					**DSI^b^**			
**Breeding lines**	**Drop-mycelium**	**Rank**	**Spray-mycelium**	**Rank**	**Iowa**	**Rank**	**Wisconsin**	**Rank**
01SSD-36	9.1	1	27.8	3	–	–	39	10
01SSD-119	10	2	29.1	5	–	–	28	5
Skylla	10	2	28	4	53.3	12	57	19
NKS 19-90	16.7	4	12.4	1	49.2	10	27	3
01SSD-20	18.2	5	38.9	11	–	–	45	14
01SSD-61	18.2	5	37.5	9	–	–	35	8
01SSD-106	25	7	45.8	19	–	–	27	3
AxN-1-68	30	8	33.3	6	36.5	4	25	2
01SSD-177	36.4	9	41.7	16	–	–	32	6
AxN-1-55	36.4	9	33.8	7	25.6	1	32	6
01SSD-150	40	11	24.3	2	–	–	44	13
LP02-221	40	11	44.9	17	62.2	19	77	25
U413038	44.4	13	40	13	36.6	5	–	–
LP02-222	45.5	14	41.3	15	72.3	25	76	24
U416019	45.5	14	35.9	8	35.8	3	–	–
LD00-1938	50	16	39.2	12	–	–	51	15
BSR101	54.5	17	45.2	18	–	–	66	22
Ohio FG3	55.6	18	48.2	22	54.2	13	20	1
U419020	57.1	19	46.8	20	38.3	7	55	18
U409014	58.3	20	49.9	23	66.3	21	51	15
AxN-2-55	60	21	38.3	10	43.7	8	43	11
U412014	60	21	76.1	34	34.8	2	36	9
U423040	62.5	23	56.6	25	45.6	9	–	–
E99279	63.6	24	61.9	27	54.9	16	43	11
NE3303	63.6	24	47.9	21	54.7	15	–	–
U409006	70	26	61.1	26	55.6	17	64	21
E99250	71.4	27	69.2	33	49.9	11	52	17
LP02-240	72.7	28	61.9	27	58.4	18	78	26
LP02-253	75	29	63	30	68.4	23	89	29
U425043	75	29	40.1	14	36.7	6	–	–
LP02-250	80	31	61.9	27	66.4	22	84	28
A2506	81.8	32	66.7	32	54.5	14	61	20
HSO-3243	83.3	33	63.3	31	62.9	20	71	23
Dwight	100	34	83.3	35	70.7	24	79	27
LD00-497	100	34	55.6	24	–	–	94	30
Mean	51.3		47.3		51.5		52.7	
LSD^0.05^	26.8		27.5		20.7		22.8	

a *Plant mortality that ranged from 0 = all plants survived to 100 = all plants dead*.

b *Disease severity index that ranged from 0 = all healthy plants with no disease to 100 = all plants killed by disease. The DSI means are based on the disease ratings of 30 plants in three replications*.

### Field evaluations

Field experiments for 35 breeding lines or varieties were carried out in Iowa and Wisconsin during the summer of 2004. The experiments were arranged in RCBD with three replications for both locations. In Iowa, single row plots of 4 m length and half meter row spacing were used. Corn was used as a wind barrier around the nursery. The plants were inoculated with sorghum seeds infested with *S. sclerotiorum* after canopy was complete at the R2 growth stage. The misting system equipped with a sensor was used to maintain leaf wetness from the day of inoculation to the end of flowering. In Wisconsin, disease nursery plots had five 5.9 m rows. Row spacing was 76 cm between border and experimental accessions and 38 cm between each experimental row to encourage a dense canopy. Common susceptible accession (Golden Harvest H2627RR) was planted in the two outer rows and experimental accessions were planted in the three middle rows. Sunflower heads were inoculated with ascospores to produce sclerotia in the disease nursery in the year preceding the soybean trial. The canopy of the soybean plants almost completely covered the row space at the R1 stage when apothecia of *S. sclerotiorum* appeared under the canopy.

Disease scoring was done in accordance with modifications of a disease severity index (DSI) described by Grau et al. (1982) at the R7 growth stage. Ten consecutive plants from each of the three experimental rows were rated on a 0–4 scale: 0 = no symptoms; 1 = lesions on lateral branches only; 2 = lesions on main stem, no wilt, and normal pod development; 3 = lesions on main stem resulting in plant death and poor pod fill; 4 = lesions on main stem resulting in plant death and no yielding pods. A DSI was calculated as: 100 ^*^ [(sum of ratings for a plot)/ [4(number of ratings classes) ^*^ 30 (number of plants rated/plot)]].

### DNA isolation

Fresh, Young, and tender leaves from the 392 F_4 : 6_ lines were collected and stored at −80°C for 2 days before lyophilization. The lyophilized tissue was ground by shaking vigorously with glass beads in a 15-ml tube with a paint shaker. The genomic DNA was extracted with the CTAB (hexadecyltrimethyl ammonium bromide) method as described by Kisha et al. (1997) and the DNA quantification was performed in a ND-1,000 Spectrophotometer (NanoDrop Technologies, Inc, Wilmington, Delware). The quality of genomic DNA was checked by gel electrophoresis in 1% agarose gel.

### Polymerase chain reaction (PCR)

A total of 132 simple sequence repeat (SSR) primer pairs were selected from the integrated soybean linkage map (Song et al., 2004; Choi et al., 2007). These SSRs included those markers that are significantly associated with and flank already reported 33 QTL for SWM resistance (retrieved from SOYBASE, http://soybase.org/). Of the 33 QTL for resistance to SWM investigated, 28 were identified by Arahana et al. (2001) in NKS 19-90 and other partially resistant cultivars, three were identified by Kim and Diers (2000) in NKS 19–90, and two were identified by Guo et al. (2008) in PI 391589A, PI 391589B, Kottman, and IA2053.The selected SSRs were then used to screen parents for polymorphism.

The PCR amplification was performed in MJ TetradTM thermal cycler (MJ Research, Waltham, MA). Total reaction volume of 15.0 μL contained 50 ng of genomic DNA, 0.3 μM each of forward and reverse primers, 0.2 mM of dATP, dCTP, dGTP, and dTTP (Sigma-Aldrich, St. Louis, MO), 3.0 mM MgCl_2_, 2.5 units of Taq polymerase, and 1.0 × PCR buffer. The PCR was performed using a regular program as follows; an initial denaturation at 95°C for 2 min, followed by 38 cycles of denaturation at 94°C for 25 s, 25 s of annealing at primer specific annealing temperature, 45 s of extension at 70°C, a final extension at 72°C for 10 min, followed by a final hold at 4°C. The PCR products were separated on 6% non-denaturing polyacrylamide gels using an electrophoresis unit DASG-400-50 (C.B.S. Scientific Co. DelMar, CA) as described by Wang et al. (2003). Ethidium bromide was used to stain the gel and PCR products were visualized under UV light, and photographed. Genotyping with SSR markers was carried out as described by Wang et al. (2003). A total of 42 polymorphic markers were then used to genotype the 392 F_4 : 6_ lines of the seven populations. For each polymorphic marker, the DNA bands were scored based on their fragment sizes on the parents.

### Statistical analyses

The PROC MIXED in SAS studio (SAS Institute Inc., 2015) with COVTEST statement was used to compute estimates of variances, standard errors (SE) associated with them, broad-sense heritability (*h*^2^), and SE associated with them for resistance to SMW in seven mapping populations. Broad-sense heritability (*h*^2^*)* estimate of each trial was calculated according to Holland et al. (2003) using the formula: $h^{2}=\frac{\sigma^{2}G}{\left[\sigma^{2}G+(\sigma^{2}GE/e)+(\sigma^{2}/re)\right]}$, where σ^*2*^*G*, σ^*2*^*GE*, and σ^*2*^ are variance components of genotype, genotype x experiment, and experimental error, respectively. The number of experiments and replications used are denoted by e and r, respectively. We employed Fisher's protected least significant differences (LSD) test at 5% significance level to perform pairwise comparisons of means for 66 PIs using LSD.test function in R (R Core Team, 2013). The function pairs.panels (psych package) in R (Revelle, 2017) was used to create scatterplot matrices and to compute Pearson's product moment correlation coefficients (r) for the breeding lines or varieties between drop-mycelium and field trials, between spray-mycelium and field trials, and between greenhouse (across inoculation techniques) and field trials. Single-marker analysis (SMA) was performed across populations and inoculation methods using QTL Cartographer 2.5_011 (Wang et al., 2007).

## Results

### Phenotypic results

All the PIs, breeding lines or varieties, and F_4 : 6_ lines showed typical symptoms and signs of SWM. Necrotic lesions and white fluffy mycelia were distinctly visible on the apical meristems and main stems. Due to the random nature of spraying in spray-mycelium inoculation, secondary infections at multiple points were distinctly visible on the whole plants, whereas disease progressed downward from the apical meristem in drop-mycelium evaluation. Disease developed and progressed very fast in susceptible plants but was arrested on the apical meristem in highly resistant plants.

The 66 PIs (Table 3) were significantly (*P* < 0.043) different from each other. PI x year interaction (*P* < 0.623) was not found significant. Year was highly significant (*P* < 0.00001). The Fisher's LSD at 5% significance was 32.9. The PMs for resistant (NKS 19–90) and susceptible (Olympus) checks are 31.3 and 72.3%, respectively. PI 361059B, FC 030233, PI 358318A, PI 506654, PI 506728, PI 427143, PI 504502, and PI 506733A consistently showed high level of resistance to SWM for both years (Table 3). These resistant PIs exhibited resistance level similar to that of NKS 19–90 in the current study.

**Table 3:** Ranking of 66 plant introductions (PIs) based on plant mortality (PM) using drop-mycelium method in our greenhouse evaluations and the disease severity index (DSI) from Hoffman et al. (2002) study.

**Plant mortality^a^**							**Disease severity index^b^**	
**Plant introduction**	**2009**	**Rank**	**2010**	**Rank**	**Across-year**	**Rank**	**DSI**	**Rank**
PI 506733A	8.3	7	30.6	2	19.4	1	21	58
PI 504502	5.6	2	37.8	3	21.7	2	18	49
PI 506654	5.6	2	47.2	10	26.4	3	13	37
PI 506728	22.9	18	30.0	1	26.4	4	23	62
PI 361059B	18.9	15	41.1	5	30.0	5	6	6
PI 358318A	19.1	17	41.7	6	30.4	6	10	21
PI 417201	6.7	5	55.6	22	31.1	7	14	40
PI 594286	0.0	1	64.4	32	32.2	9	20	57
FC 030233	26.7	22	41.7	6	34.2	10	7	13
PI 416805	8.3	7	63.9	31	36.1	11	9	17
PI 427143	31.1	31	41.7	6	36.4	12	7	13
PI 189919	16.7	14	57.8	23	37.2	13	7	13
PI 196157	9.5	9	65.6	35	37.5	14	16	43
PI 507222	26.2	20	50.0	13	38.1	15	18	49
PI 184042	11.1	11	67.2	41	39.2	16	19	55
PI 548539	27.8	25	52.8	16	40.3	17	16	43
PI 132207	42.2	49	40.0	4	41.1	18	0	1
PI 507353	28.6	27	54.4	19	41.5	19	4	3
PI 506892	9.5	9	75.6	50	42.5	20	15	42
PI 561367	13.3	13	75.0	47	44.2	21	6	6
PI 506784	5.6	2	83.3	55	44.4	22	11	29
PI 548407	24.5	19	65.6	36	45.0	23	6	6
PI 232996	36.1	41	54.2	18	45.1	24	24	63
PI 081775	35.3	38	55.6	20	45.4	25	14	40
PI 438267	35.6	39	55.6	20	45.6	26	18	49
PI 507352	26.7	22	65.0	34	45.8	27	3	2
PI 416776	33.3	33	58.3	24	45.8	28	10	21
PI 243547	32.2	32	60.0	26	46.1	29	4	3
PI 549066	18.9	15	75.0	47	46.9	30	10	21
PI 153259	50.0	52	45.6	9	47.8	31	21	58
PI 567157A	6.7	5	88.9	63	47.8	32	6	6
PI 506652	26.5	21	70.0	44	48.3	33	11	29
PI 091733	45.0	50	52.8	16	48.9	34	11	29
PI 404180	38.0	44	60.5	27	49.2	35	18	49
PI 398637	31.0	30	68.3	42	49.6	36	17	46
PI 561353	36.7	42	63.3	30	50.0	37	6	6
PI 153282	35.7	40	65.7	37	50.7	38	6	6
PI 548312	37.8	43	64.4	32	51.1	39	6	6
PI 561345	52.2	55	50.0	13	51.1	40	13	37
PI 548404	41.7	46	66.7	38	54.2	41	16	43
PI 594289	28.3	26	80.6	53	54.4	42	30	66
PI 417245	26.7	22	83.3	55	55.0	43	21	58
PI 548380	41.7	46	68.9	43	55.3	44	19	55
PI 189899	50.0	52	61.1	28	55.6	45	10	21
PI 578501	50.0	52	61.1	28	55.6	45	12	35
PI 567650B	33.3	33	81.1	54	57.2	47	8	16
PI 417533	66.7	65	50.0	11	58.3	48	11	29
PI 189896	33.3	33	83.3	55	58.3	49	10	21
PI 506868	33.3	33	83.3	55	58.3	49	17	46
PI 506519	66.7	65	50.6	15	58.6	51	26	65
PI 153316	60.0	59	58.3	24	59.2	52	11	29
PI 417449	30.0	28	88.9	63	59.4	53	9	17
PI 391589B	34.3	37	86.7	60	60.5	54	5	5
PI 437764	46.0	51	76.4	52	61.2	55	10	21
PI 561284	30.0	28	94.4	65	62.2	56	11	29
PI 189931	60.0	60	66.7	38	63.3	57	17	46
PI 229324	41.9	48	86.7	60	64.3	58	12	35
PI 437072	57.9	58	72.2	45	65.1	59	13	37
PI 189861	66.7	65	66.7	38	66.7	60	21	58
PI 437527	53.2	56	83.3	55	68.2	61	9	17
PI 423818	38.9	45	100.0	67	69.4	62	24	63
PI 291319B	63.9	63	75.6	50	69.7	64	9	17
PI 561331	54.8	57	86.7	60	70.7	65	18	49
PI 417507	70.0	68	75.0	47	72.5	66	10	21
PI 567721	63.3	62	94.4	65	78.9	67	34	67
PI 548354	62.8	61	100.0	67	81.4	68	18	49
NKS 19-90^®^	12.5	12	50.0	11	31.3	8	10	21
Olympus (S)	66.7	64	77.8	46	72.3	63		
Mean	34.3		65.3		49.8		13.2	
STDEV	18.6		16.6		13.7		6.7	
CV (%) LSD^0.05^	54.2		25.5		27.5 32.9		50.8	

a *Plant mortality that ranged from 0 = all plants survived to 100 = all plants dead*.

b *Disease severity index that ranged from 0 = all healthy plants with no disease to 100 = all plants killed by disease. The DSI means are based on the disease ratings of 30 plants in two replications according to (Hoffman et al., 2002)*.

**Figure 1:**
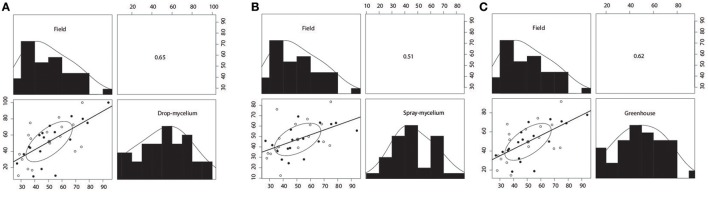
Scatterplot matrices for reaction of 35 breeding lines or varieties to soybean white mold (SWM) between drop-mycelium inoculation in greenhouse and field performance **(A)**, between spray-mycelium in greenhouse and field performance **(B)**, and between greenhouse (across inoculation methods), and field performance **(C)**. Pearson's product-moment correlations are shown in the upper panel. Plant mortality (PM) was assessed in greenhouse evaluations (drop- and spray-mycelium), whereas disease severity index (DSI) was assessed in field trials (across Iowa and Wisconsin). Correlation coefficients were significant at 0.01 level for spray-mycelium vs. field performance, while correlation coefficients were significant at 0.001 level for drop-mycelium vs. field performance and greenhouse (across inoculation methods) vs. field performance.

Plant mortalities for the 35 breeding lines or varieties ranged from 9.1 to 100% and 11.1 to 69.2% for drop- mycelium and spray-mycelium, respectively, and the PM means for the two greenhouse inoculation methods were similar (51.3 and 47.3, respectively). Similarly, the DSI means for the two field trials were very similar (51.5 and 52.7 respectively for Iowa and Wisconsin) and ranged from 25.6 to 72.3 and 20 to 94 in Iowa and Wisconsin field trials, respectively. The 35 soybean breeding lines or varieties were significantly different (*P* < 0.05) for resistance to SWM in both greenhouse and field trials (Table 2). LSDs for 35 breeding lines or varieties were 26.8, 27.5, 20.7, and 22.8, respectively. The resistant check NKS 19–90 exhibited moderate to high level of resistance (11% PM in spray-mycelium to 57% DSI in Wisconsin field trial) for different evaluation methods. However, Skylla, a proven partially resistant cultivar developed from NKS 19–90, expressed resistance level comparable to its progenitor NKS 19–90 in all evaluations. The correlation coefficient (r) between the two greenhouse evaluation methods was 0.80 (*P* < 0.00005) (Figure 1A). Correlation coefficients between PM under drop-mycelium (Figure 1A) and spray-mycelium (Figure 1B) inoculations in greenhouse and DSI in field trials were 0.65 (*P* < 0.0001) and 0.51 (*p* < 0.0018), respectively. Similarly, correlation coefficient between PM in greenhouse (across inoculation methods) and DSI in field trials was 0.62 (*P* < 0.0001) (Figure 1C).

There was significant genetic variability for plant mortality under inoculations with *S. sclerotiorum* in all populations but populations 5, 6, and 7 (Table 1). The *h*^2^ estimates for plant mortality under inoculations with *S. sclerotiorum* ranged from 0.39 to 0.66 (Table 1). The mean PMs for NKS 19–90 (resistant check) and Olympus (susceptible check) were 33.5 and 43.5%, respectively. The mean PMs for PI 437764, PI 548312, PI 153259, and PI 548404 were 57.5, 24.6, 62, and 61%, respectively. The PMs for Skylla (known resistant cultivar) and E00290 (known susceptible cultivar) were 30 and 73%, respectively (Table 1).

### Genotypic results

Altogether, 42 (~32%, Supplemental File 1) of the 132 SSR primer pairs tested were polymorphic in at least in one of the seven mapping populations and were distributed over 15 linkage groups (LGs) of soybean consensus map (Song et al., 2004). Of the 42 polymorphic markers, five (Sat_267, Satt619, Satt571, Satt651, Sat_244) showed polymorphism across all parental combinations and the remainders were polymorphic in one or more populations (Supplemental File 1).

A total of 11 SSRs (Table 4) that belonged to seven chromosomes, showed significant association with SWM resistance at least at 1% significance. These SSRs correspond to 10 QTL reported in prior studies. Total phenotypic variance explained by these SSRs ranged from 1.8 to 15.8%. Of the 11 SSRs confirmed, six SSRs (Satt478, Satt153, Satt243, Satt154, Satt571, and Sat_342) explained less than 5% of the total phenotypic variance each. The remaining SSRs (Satt523, Satt135, Satt186, Sat_199, and Satt175) explained from 7 to 15.8% of the total phenotypic variance. Together, these SSRs (Satt523, Satt135, Satt186, Sat_199, and Satt175) that encompassed 4 chromosomes (19, 17, 8, and 7), explained over 56% of the total phenotypic variance, leaving rest of the variations unexplained (Table 4).

**Table 4:** The SSR markers significantly associated with QTL controlling soybean white mold resistance as detected by single marker analysis across populations and inoculation methods.

**LG (chromosome)**	**Marker**	**Position (cM)^†^**	**Pr**	***R*^2^‡^^**	**References**
M (7)	Satt175	61.93	^****^	7.0	2^b^
A2 (8)	Sat_199	70.95	^****^	15.8	1^a^
O (10)	Satt478	66.01	^**^	2.5	1
O (10)	Satt153	106.32	^****^	3.2	1
O (10)	Satt243	107.30	^**^	2.0	1
B2 (14)	Sat_342	15.5	^**^	1.8	1
D2 (17)	Satt135	25.48	^****^	11.3	1
D2 (17)	Satt154	46.76	^***^	4.0	1
D2 (17)	Satt186	92.22	^****^	14.2	1
L (19)	Satt523	25.56	^****^	8.5	1
I (20)	Satt571	14.97	^**^	2.3	1

**, ***, **** Significant at 1, 0.1, and 0.01% levels, respectively.

† *Map position per https://soybase.org/BARCSOYSSR/index.php*.

‡ *Total phenotypic variance (%) explained by the marker genotype*.

a *Arahana et al. (2001)*.

b *Guo et al. (2008)*.

## Discussion

The PIs are major sources of pest resistance (Shoener and Fehr, 1979). Attempts were made to identify soybean germplasm that provide resistance to SWM. No qualitative resistance has been detected for SWM resistance yet (Nelson et al., 1991; Kim et al., 1999; Hartman et al., 2000; Hoffman et al., 2002; Guo et al., 2008). In an attempt to identify soybean PIs resistant to *S. sclerotiorum*, Hoffman et al. (2002) screened about 6,520 soybean PIs in different locations of the US and Canada both in the field as well as in greenhouse conditions. A total of 68 PIs were selected as resistance sources. However, these 68 PIs expressed different levels of resistance to SWM in the field conditions. Drop-mycelium inoculation method in the current study was also able to discriminate among these PIs based on their reactions to SWM. PI 361059B, FC 030233, PI 427143, PI 189919, PI 567650B, PI 358318A, and PI 416805 expressed resistance level similar to that of NKS 19–90 for mean damage in both our study and Hoffman et al. (2002). These PIs could be valuable germplasm in soybean breeding programs that aim at enhancing breeding materials for SWM resistance. There were some PIs such as PI 291319B, PI 417507, and PI 437527 that expressed high level of resistance in Hoffman et al. (2002). However, these PIs expressed low level of resistance in the current study (Table 3). The disease escape mechanism could have played some roles for field resistance in Hoffman et al. (2002). The discrepancy in level of resistance to SWM between greenhouse and field trials could also be attributed to disease screening carried out at different plant stages (V3 vs. reproductive stages) because maturity confers more resistance to SWM (Chun et al., 1987; Moellers et al., 2017).

Field tests for these PIs by Hoffman et al. (2002) were performed in eight environments in RCBD with two to four replications at each location. Three types of inoculum (inoculum in soil from previous crops, sclerotia obtained from harvested common beans spread onto soil surface, and ground grain of wheat, sorghum, or oat colonized with *S. sclerotiorum* mycelium) were used for field inoculations. The DSI was measured on a scale of 0–3 at R7 stage from 30 randomly selected plants. In the current study, greenhouse test for 66 PIs were performed according to Chen and Wang (2005), who described greenhouse-based drop-mycelium method as more efficient, reliable, and cost-effective than cut-petiole method, a commonly accepted inoculation technique. In addition, drop-mycelium method is scalable for a large-scale SWM resistance evaluation. Above all, drop-mycelium method could be used to screen soybean plants in early (V3) growth stage, obviating the need to grow plants until reproductive stages. Thus, this method could help reduce the dependence on natural disease pressure to evaluate soybean germplasm in field trials. Consequently, this method could serve as a substitute for field screening of SWM resistance.

In greenhouse evaluations, AxN-1-55, a registered elite germplasm in 2006 (Diers et al., 2006), exhibited PM intermediate (35.1) between its partially resistant parents, NKS 19–90 (14.6) and A2506 (57.8). However, AxN-1-55 had field resistance levels higher (28.8) than NKS 19–90 (38.1) and A2506 (74.3) agreeing with previous evaluations made by Diers et al. (2006) in 11 environments. Generally, 01SSD breeding lines (Table 1) along with AxN-1-55 and Ax-N-1-68 showed stable and high level of resistance across all trials corroborating results by other authors (Hoffman et al., 2002; Chen and Wang, 2005). Thus, these breeding lines could serve as valuable germplasm for resistance to SWM in soybean breeding programs. BSR 101 could be used as a source of partial resistance to SWM in breeding programs. Dwight could be used as a susceptible line to develop populations for SWM resistance QTL studies.

Greenhouse or laboratory testing of soybean resistance to white mold has been compared with field trial results in prior studies. However, strong correlations between field and greenhouse or laboratory evaluations were not reported. Nelson et al. (1991) argued that greenhouse or laboratory tests were not reliable to predict field resistance to SWM. Moellers et al. (2017) have reported correlation coefficients between greenhouse and field evaluations that ranged from 0.12 to 0.17 using cut-petiole inoculation method and suggested that an increased correlation coefficient could be achieved if plants were inoculated at more mature stages in greenhouse evaluations. The discrepancies in correlation coefficients between Moellers et al. (2017) and the current study could be ascribable to different inoculation techniques used. In the current study, the moderate and significant correlation coefficient between greenhouse (across different methods) and field evaluations (*r* = 0.62) for reactions of 35 breeding lines or varieties suggested that field resistance to SWM could be predicted by greenhouse evaluation methods (Kandel, 2011). In essence, due to the highly polygenic nature of inheritance and low heritability of the trait, highly positive correlation coefficients between greenhouse and field trials are very difficult to achieve for SWM resistance. The findings in this study suggest that spray-mycelium and drop-mycelium methods could be viable greenhouse evaluation methods to predict the field resistance to SWM. Further, these are *in vivo* inoculation methods and thus do not require wounding of the plants for testing. Therefore, the induced resistance that plant may develop after wounding could be avoided.

Overall, our results suggest that drop-mycelium method is more informative than spray-mycelium method in predicting field resistance to SWM. However, spray-mycelium method is more useful than drop-mycelium method if the objectives of a breeding program are to screen germplasm in a large-scale and to identify a few highly resistant genotypes because it is more efficient and severe than the drop-mycelium technique.

The *h*^2^ estimates in our seven mapping populations closely agree with prior studies (Kim and Diers, 2000; Guo et al., 2008; Vuong et al., 2008). The estimates of genotypic variance (σ^2^G) for population 5, 6, and 7 in the current study were not significantly different from zero based on *Z*-scores at 5% significance level. However, it should not imply that genetic components are necessarily negligible, but rather accurate estimates of *h*^2^, which vary depending on the population size, number of replications, and allele frequency in the population, are difficult to achieve (Roff, 1990). The SE of *h*^2^ are large in the current study probably due to small population sizes. However, it is evident from prior studies and the current study that SWM resistance has low to moderate *h*^2^ estimate.

Because the number of individuals in each of the seven biparental populations (Table 1) were small, QTL analysis was done across populations (a total of 392 individuals). This allowed us more power to detect SSRs significantly associated with QTL and estimate their effects than if QTL analysis was performed for individual populations. Because QTL studies especially SMA in bi-parental populations present large confidence intervals, the significant markers could be loosely linked with the trait, and the QTL may not lie between flanking markers. Of the 11 SSRs (corresponding to 10 QTL) confirmed in this study, nine QTL were reported by Arahana et al. (2001) with SMA in five populations derived by crossing Williams 82, a susceptible cultivar, with five partially resistance cultivars: NKS 19-90, Corsoy 79, Dassel, DSR173, and Vinton 81. The significant SSR (Table 4) on LG M (Chromosome 7) was reported by Guo et al. (2008) in 94 F_2_-derived lines from a cross between PI 391589B (partially resistant), and IA 2053 (moderately susceptible). However, no significant SSRs were detected in our study for the QTL reported by Kim and Diers (2000). This could be due to various reasons such as use of different Sclerotinia isolates and/or different genetic backgrounds of parents and/or different disease inoculation techniques in two studies. More importantly, the alleles for two of the three QTL detected by Kim and Diers (2000) that were significantly associated with SWM resistance were also associated with disease klendusity. Because this study was conducted in controlled greenhouse conditions and efforts were made to ensure upright architecture of plants using upside down plastic cups, the significant markers we detected should not co-localize with disease klendusity. The phenotypic variance explained by each QTL for SWM resistance was less than 16% agreeing with most results from prior studies; although Huynh et al. (2010), Iquira et al. (2015), and Moellers et al. (2017) reported some major QTL for resistance to SWM. The presence of many QTL with minor effects confirms that soybean resistance to white mold is a complex trait.

The markers Satt153 and Satt243 (~45.9 Mbp in chromosome 10) significantly associated to SWM probably detected the same QTL reported by Arahana et al. (2001). Moellers et al. (2017) also detected a significant SNP at 47.6 Mbp on chromosome 10 using GWAS and the gene containing the SNP (Glyma.10g247900) was proposed as candidate gene for that QTL. We found three SSRs (Satt135, Satt154, and Satt186) on three distinct genomic regions of chromosome 17 that were significantly associated with SWM resistance QTL, validating QTL reported in Arahana et al. (2001) and in McCaghey et al. (2017). Similarly, the significant marker Sat_199 (at 15.14 Mbp) on chromosome 8 could be related to the QTL reported by Arahana et al. (2001) and Moellers et al. (2017) at 17.5 Mbp on chromosome 8. Finally, significant marker Satt175 (at 15.3 Mbp) on chromosome 7 would validate the QTL reported by Guo et al. (2008) and Sebastian et al. (2010). Thus, validation of QTL in different genetic backgrounds in these studies further provides evidence that these QTL are stable and thus could be transferred into elite soybean germplasm. Also, this suggests that these greenhouse inoculation techniques could be used in future studies to study SWM resistance QTL.

QTL studies have revealed that SWM resistance was controlled by many genetic loci with small effect and their interactions (Kim and Diers, 2000; Arahana et al., 2001; Guo et al., 2008; Moellers et al., 2017). However, candidate QTL have been validated on chromosome 15 using SNP and cleaved amplified polymorphic sequence markers (Bastien et al., 2014). Information on validated SSRs that correspond to major QTL from chromosome 8, 17, and 19 in our study coupled with information from Bastien et al. (2014) could facilitate introgression of these QTL alleles into commercial cultivars. In fact, pyramiding of resistant loci for SWM resistance from different resistant sources has been proved feasible. For instance, an increased level of resistance in AxN-1-55 to SWM has been achieved by combining resistance from two partially resistant cultivars, NKS 19-90 and A2506 because AxN-1-55 has higher resistance to the disease than either parents (Diers et al., 2006). Information on the validated SSRs for SWM resistance in the current study should thus help soybean breeders in that direction.

Because there were a few polymorphic markers per linkage group in the current study, locations and effects of QTL could not be determined precisely. Thus, marker coverage and population size should be increased in future studies to precisely locate the validated QTL and accurately determine their effects. With the dense marker coverage around the QTL, marker-assisted selection (MAS) could be used to introgress alleles of these QTL into commercial cultivars. However, as prior QTL studies (Bastien et al., 2014; Iquira et al., 2015; Zhao et al., 2015; McCaghey et al., 2017; Moellers et al., 2017; Wei et al., 2017) have reported that many small effect QTL controlled SWM resistance, genomic selection would be very crucial to improve soybean cultivars for resistance to SWM given its complex and quantitative inheritance. The five new PIs used in this study harbor the QTL identified by Arahana et al. (2001) and Guo et al. (2008). Thus, these PIs could be used as sources of SWM resistance in soybean breeding programs.

## Author contributions

DW developed the study and designed the experiments. RK and CC conducted the greenhouse experiments. CG, AD, JL, and YW conducted the field trials. RK conducted the genotyping, data analysis and wrote the manuscript. CC contributed to the drafting of the manuscript. All authors reviewed and approved the manuscript. DW is the corresponding author and the PI of this project.

### Conflict of interest statement

The authors declare that the research was conducted in the absence of any commercial or financial relationships that could be construed as a potential conflict of interest.

## Supplementary material

Supplemental File 1 Primer combinations and PCR conditions for 42 polymorphic SSR markers.
